# Perceptions and Expectations of Patients with Lung Cancer and Melanoma about the Telenursing Approach: A Phenomenological Study

**DOI:** 10.3390/nursrep14040198

**Published:** 2024-09-27

**Authors:** Aurora De Leo, Sara Dionisi, Alessandro Spano, Laura Iacorossi, Gloria Liquori, Noemi Giannetta, Emanuele Di Simone, Paola Presta, Fabrizio Petrone, Marco Di Muzio, Nicolò Panattoni

**Affiliations:** 1 Department of Biomedicine and Prevention, University of Rome Tor Vergata, 00133 Rome, Italy; aurora.deleo@ifo.it (A.D.L.); alessandro.spano@ifo.it (A.S.); gloria.liquori@uniroma1.it (G.L.); 2 Nursing Research Unit IFO, IRCCS Istituti Fisioterapici Ospitalieri, 00144 Rome, Italy; 3 Nursing, Technical, Rehabilitation Department, DaTeR Azienda Unità Sanitaria Locale di Bologna, 40124 Bologna, Italy; sara.dionisi@uniroma1.it; 4 Department of Life, Health and Health Professions Sciences, Link Campus University, 00165 Rome, Italy; l.iacorossi@unilink.it; 5 Departmental Faculty of Medicine, UniCamillus, 00131 Rome, Italy; noemi.giannetta@unicamillus.org; 6 Department of Public Health and Infectious Diseases, Sapienza University of Rome, 00185 Rome, Italy; nicolo.panattoni@uniroma1.it; 7 Nursing, Technical, Rehabilitation, Assistance and Research Direction, IRCCS Istituti Fisioterapici Ospitalieri, IFO, 00144 Rome, Italy; paola.presta@ifo.it (P.P.); fabrizio.petrone@ifo.it (F.P.); 8 Department of Clinical and Molecular Medicine, Sapienza University of Rome, 00185 Rome, Italy; marco.dimuzio@uniroma1.it

**Keywords:** expectations, lung cancer, melanoma, neoplasms, nurse, patient, perceptions, qualitative research, telenursing

## Abstract

Background: Telenursing could improve continuity of care in patients with cancer. This study aims to explore the expectations and perceptions of patients with lung cancer and melanoma toward telenursing. Methods: A descriptive qualitative study using a phenomenological approach was conducted on a convenience sampling of twenty patients aged 18 years or over from a Cancer Center. With the consent of patients and the relevant Ethics Committee, in-depth open-ended face-to-face interviews were conducted until data saturation. The phenomenon’s essence was achieved through themes emerging from the qualitative data analysis. Results: Patients’ perceptions and expectations were related to areas explored by a general theme on the nurse–patient relationship’s importance. Four themes and eleven sub-themes were focused on misconceptions about lack of use, patients’ potential and fears, the home as a place of care, and the caring relationship. Fifteen patients perceived the internet as a chaotic “bubble”. Conclusions: Despite the lack of previous use, patients consider telenursing positively as “a bridge between home and care”, especially in the advanced stages of the disease. They highlighted strengths and weaknesses of telenursing, such as having “someone for you”, connection, fear of psychological addiction, loss of privacy, and lack of empathy. This study was not registered.

## 1. Introduction

Telenursing is an offshoot of eHealth and a valuable nursing approach in primary and cancer care to improve patient-centered care, self-care skills, patient well-being, quality of life, continuity, and access to care [1,2]. In particular, telenursing refers to the set of tools and interventions that use information and communication technologies to improve data transmission, patient treatment pathways, access and quality of care [1,3]. Even in oncology care, the main objectives of telenursing concern the strengthening of traditional clinical practice, improvement in the communication and transmission of information between the patient and care team, continuity of care, education, and medication adherence [4]. In recent years, several oncology studies have been conducted using different telenursing interventions and tools to improve patient support in cancer care. In particular, several studies have investigated the effectiveness of the telenursing approach in patient education interventions, in addition to or as an alternative to traditional face-to-face care, with promising results in young and elderly people [5,6,7]. Although eHealth and telenursing have been used in cancer care, their implementation in clinical pathways has not fully considered patients’ needs, satisfaction, and perspectives [8]. In this regard, nurses should ensure that this approach is based on patients’ values and needs, does not increase inequalities in access to care and caring, and, in particular, allows for the preservation of the quality of the nurse–patient relationship, “the core” of nursing care [3]. In this regard, some studies suggest that one of the main barriers to telenursing for nurses and patients is the fear of losing the nurse–patient relationship [9,10]. 

Given the increased prevalence and incidence of lung cancer and melanoma observed worldwide in recent years [11], this qualitative study aims to explore the expectations and perceptions of patients with lung cancer and melanoma about telenursing. Improving knowledge of this population’s perceptions and expectations of telenursing could improve the design, dissemination, and use of these tools among stakeholders.

## 2. Materials and Methods

Qualitative research is one of the best approaches to better understand patients’ knowledge, perspectives, and barriers to issues related to their health and care pathways [12]. In particular, the phenomenological approach appears to be the best model for exploring patients’ in-depth perceptions of care [13], capturing the global experience and the essence of a phenomenon by thematizing it. In this approach, data are collected through open, face-to-face interviews to capture subjective feelings through the interviewees’ stories [14]. Through the analysis, the researcher captures the essence of the experience, which the participants could not achieve independently, giving them back the meaning of the experience itself, according to Husserl’s perspective [15]. The phenomenon becomes the product of the experience of the subject who experiences it and of the researcher who can grasp its essence through the unique interdependent interaction between the object (the phenomenon) and the subject related to it. 

### 2.1. Study Design 

A qualitative, monocentric, descriptive study [16] using the phenomenological approach following Husserl’s perspective [15] was conducted to explore the expectations and perceptions of patients diagnosed with melanoma and lung cancer about telenursing. Through in-depth, open-ended face-to-face interviews, nurses can gain a deeper knowledge of the patient’s perspective on the phenomenon [15]. The COREQ guidelines (Consolidated Criteria for Reporting Qualitative Research) [17] (Table S1 in the Supplementary Materials) and Lincoln and Guba’s criteria [18] enhanced this study’s consistency, quality, and rigor. Aspects of interest that might emerge from the results of the thematic analysis can be addressed later through different study designs or methodological approaches such as quantitative research.

Table S1. COREQ (Consolidated Criteria for Reporting Qualitative Research) Checklist.

### 2.2. Participant Recruitment

A convenience sampling method was used to increase the collection of patients’ expectations and perceptions of telenursing [19,20]. Specifically, a selection of patients suffering from lung cancer and melanoma was made to improve the generalizability of the results. Due to the trouble of accessing patients with lung cancer and melanoma with experience of the phenomenon within the study Cancer Center, it was not possible to implement purposive sampling. In order to collect rich and comprehensive information to maximize understanding of the phenomenon, convenience sampling was chosen by the researchers. The subject and aim of this study were presented to all eligible patients affected by melanoma and lung cancer, who were approached face to face by two nurses from an Italian National Cancer Centre in September 2023. Before the interviews, the enrolled patients signed a specific informed consent form to allow the researchers to collect, record, analyze, and disseminate the data. To promote the appropriateness of the sampling strategy, the following criteria were used for enrolment [21]. Inclusion criteria: Patients with melanoma and lung cancer, aged ≥ 18 years, able to understand and speak Italian, able to sign a written informed consent, and willing to comply with the study procedures. Exclusion criteria: Patients with cognitive impairment, psychiatric disorders, and poor compliance with study procedures.

### 2.3. Data Collection

Following the phenomenological approach [15] to gaining an in-depth understanding of the topic, nurses and patients conducted open-ended one-on-one interviews lasting up to 20 min until data saturation [19,20]. The interview included six questions built according to the key topics [22]. Before the interviews, nurses introduced patients to the topic and the research questions (Table 1) based on previous studies [23,24], along with the meaning of the term telenursing and what it refers to in cancer care. The interviews were audio-recorded and then transcribed into tables and grids to assess the achievement of data saturation, identified by the redundancy of emerging features, observations, and lack of new concepts and information from the interviews [19,20]. This key point overlaps with the redundancy of the information emerging from the latest interviews. The researchers conducted the data collection and analysis simultaneously, coding and aggregating the information obtained until the emerging categorizations were repeated (data saturation). Data saturation is a key element of qualitative research. Indeed, the redundancy of the data reasonably allows the researcher to believe that they have not overlooked information that could consequently alter the research results [25]. This approach resulted in data saturation and an optimal sample size [19,20]. Before the interviews, patients were asked to use free detailed descriptions, metaphors, and examples from everyday life to enrich the descriptions. In addition to narrative reports, sociodemographic and clinical information was collected.

### 2.4. Data Analysis

The data analysis adopted Giorgi’s phenomenological approach [26] and the inductive process of the content analysis method [27]. Specifically, the data analysis used the five stages within Giorgi’s descriptive phenomenological approach [26]: data collection (interviews); reading the data; dividing the data into parts; organizing the data in a disciplinary perspective; synthesizing the data to express the structure of the phenomenon.

One nurse collected the interviews, which were simultaneously transcribed word for word (verbatim) with pauses, silences, and gestures by another research nurse expert in the local language. In the second stage, the nurses reread the transcription to obtain the overall meaning and the person’s point of view without preconceptions and without aggregating the data. In the third phase, nurses reread the text in search of the primary “units of meaning” concepts with their independent meaning [26]. The “units of meaning” were then reorganized from a disciplinary perspective and found to be consistent with the clinical perspective. Finally, in the abstraction phase, similar units of meaning were grouped into macro-categories to define the phenomenon’s essence and explained through topic-related themes and sub-themes. The data analysis was carried out by two nurses and supervised by two senior nurses experienced in qualitative research. The nurses who conducted the interviews were familiar with the research topic, unlike the supervisors who were not. A discussion with the researchers was held following this study’s aims to capture the essence of the phenomenon [19] about patients’ perceptions and expectations of telenursing. No qualitative analysis software was used.

### 2.5. Ethical Considerations

This study was approved by the local Ethics Committee (RS06/IRE/23 RS/05(2854)) on 5 July 2023 and was conducted in line with the Declaration of Helsinki [28]. Patients who met the inclusion criteria were informed about the aims, objectives, and procedures of this study; the data processing and analysis procedures; and their right to refuse or withdraw from this study at any time without affecting their care pathway and quality of care. Before the interviews, patients signed a specific and detailed informed consent form, and the nurses established a positive, trusting relationship with the participants. After each interview, the nurse debriefed with the interviewee to assess the patient’s exposure to the intervention. Patient participation was voluntary. The data were presented in an aggregated and pseudo-anonymized form.

### 2.6. Rigor and Reflexivity

The researchers used a reflexive approach to reduce bias by reflecting on their judgements and biases during the data collection and analysis. Lincoln and Guba’s criteria [18] were used to ensure the trustworthiness of the results through the following criteria: credibility, dependability, confirmability, and transferability. The requirements were achieved through the following tools: persistent observation and data collection until data saturation; the triangulation of sources, methods, and researchers; constant interaction between members of the research team; an analysis of contrary cases; discussion with colleagues; peer debriefing; peer review; audit tests with researchers external to this study, experienced in qualitative research and inexperienced in the research topic; diaries. Two researchers independently collected, transcribed, and coded data to enhance credibility, and reach descriptions were used to achieve transferability.

## 3. Results

### 3.1. Sociodemographic and Clinical Characteristics

Twenty patients with melanoma and lung cancer were included in this study through convenience sampling. Table 2 shows the main sociodemographic and clinical characteristics.

In total, 65% of patients had at least a high school education; most reported having a job (55%), being married (65%), and living with their partner (75%). Most of the sample (55%) had melanoma. The most common previous treatment was surgery, and 75% of the sample were currently receiving medical cancer treatment. With the exception of one patient, the nine nonsurgical patients had lung cancer. A total of 11 patients had not received any previous medical treatment (chemotherapy or radiotherapy). Seven of them have had only surgery and four were at the beginning of their clinical course. The latter were all suffering from lung cancer. Ten melanoma patients were on immunotherapy treatment at the time of data collection. The four patients undergoing chemotherapy had lung cancer. No patients had prior experience of telenursing (0%). No interviews were repeated; three patients refused to participate due to lack of time, and two due to feeling unwell. No attrition was observed.

### 3.2. Phenomenological Thematic Analysis: Themes and Sub-Themes

**Figure 1:**
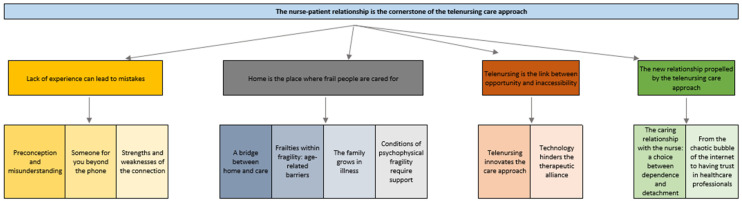
Themes and sub-themes.

Following the content analysis and the inductive method, one general theme, four themes, and eleven sub-themes emerged from the phenomenological analysis [15,26,27] (Figure 1). Following Newberry [29], Table 3 and Table 4 show the patient and interview information and the prevalence of the themes that emerged from each interview, allowing the reader to access the raw data and better understand the authors’ analytical process. Finally, with the same intention, Table S2 in the Supplementary Materials shows quotations from each theme and sub-theme. The themes were extracted from the verbal transcription of the interviews. For each patient, an assigned code (P1-P20), gender (F = female and M = male), and age were given in brackets after the sentence, i.e., (P20, M, 46 years old).

### 3.3. General Theme: The Nurse–Patient Relationship Is the Cornerstone of the Telenursing Care Approach

The nurse–patient relationship was the dominant, transversal, and unifying element in the four emerging themes of this study. Patients saw telenursing as a tool to improve the communication and information flow of the care team with the support of nurses. The caring relationship based on trust only reassures patients that it is possible to maintain care and professional skills, even at a distance.

Table 5 shows the prevalence of the themes and sub-themes by cancer, age, gender, previous surgery, and ongoing treatment.

#### 3.3.1. Theme 1: Lack of Experience Can Lead to Mistakes

Patients’ lack of direct experience with telenursing leads to misunderstandings about the aim of telenursing. Nine patients said that they had never heard of telenursing, five gave a literal translation of the term, and none had a clear idea of what it was. Four patients talked about telenursing as a futuristic approach, like something for American nurses (one patient): “Telenursing makes me think of American nurses. I think it could be a very beneficial service but I’m not sure what it is. I don’t know, but I think it’s suitable for patients”. (P18, D, 75). Five patients thought telenursing was a tool to postpone visits to the doctor, and nine identified it as the use of the telephone. Patients felt that telenursing could help them receive immediate help from nurses, thereby relieving doctors of an excessive workload (four patients): “The doctor is only sometimes available for a problem, so you may always have someone to call. In short, the nurse can help you explain more things than the doctor. The doctor is always a little busier, right? No one discriminates against the nurse, but the nurse lives in the hospital daily, while the doctor is there for 5/6 h and then goes home”. (P8, M, 59). Patients emphasize the speed of response to care needs facilitated by connectivity, but the lack of it is also a source of concern for six of them. “Sometimes, I need a better connection. Very often, the line, despite having been improved, is weak; sometimes, the connection might be more optimal. Having an optimal connection to the Internet, that’s all” (P17, M, 57).

#### 3.3.2. Theme 2: Home Is the Place Where Frail People Are Cared for

The home becomes a safe place where people can positively face the changes brought by the illness: “The home becomes a place where these fragile people can feel better/good. It is possible to work together with the hospital in particularly fragile situations, even from home and remotely, avoiding conducting fragile patients from home to the hospital. Sometimes the nurse’s advice, even remotely, has the same effect as doing it in person, but the fragile patient remains in the safety of his home (P1, M, 48)”. Five patients see telenursing as a bridge between home and hospital care to improve the patient’s well-being and safety and increase family support. Even in the home’s safety, the disease has a hard impact on patients and their families, overwhelming the psychophysical and social sphere of the patient and requiring support from the family, as highlighted in eight interviews: “The disease makes you alone, even within your family. Sometimes, many people are close to you, but they don’t know how to help you. An external person could also help family members who don’t know what or how to say or how to behave” (P14, F, 44). For six patients, the family’s response may be to grow with the patient or to ascribe care to others, losing the challenge and becoming overwhelmed by the burden of care. Seven patients stated that ageing could be an additional barrier to the use of telenursing in frail cancer patients: “It will undoubtedly be a little more problematic for older people, but they have to get used to everything a little, perhaps with the help of a relative or someone who assists them” (P10, M, 61).

#### 3.3.3. Theme 3: Telenursing Is the Link between Opportunity and Inaccessibility

All of the patients needed a clearer understanding of telenursing. Despite the lack of knowledge, they believe that it could improve patient care and closeness with the care team, help obtain information, and renew the care relationship: “Constantly interacting immediately with people who can give answers is undoubtedly a positive thing. Nowadays, interactive meetings remotely are almost the same as meeting physically, so I find this to be a positive thing” (P10, M, 61); “It’s an extra weapon…. That bow is really heavy, instead we need to lighten it…(With telenursing) we all take responsibility for the care, and in any case, we end up having much less impact on the hospital, transportation, parking and everything, because the care moves to the person, and the nurse arrives in people’s homes”. (P1, M, 48). At the same time, however, the fear that technology could replace face-to-face care casts a shadow over this care approach (four patients), as suggested by the sub-theme “Technology hinders the therapeutic alliance”. In this regard, four patients believe that the caring relationship with nurses requires empathy and that only closeness can make this possible, expressing their mistrust of these tools: “I have some doubts caused by my perception of the nature of the nurse’s work compared to that of the doctor. I never expect great empathy from the doctor, I hope it from a nurse, precisely on a human level and the telematic relationship is cold, by nature. It must not become a replacement. Direct contact with patients must never be lacking in patient care…Even being Italian could be a cultural problem; that is, if this service allows you to save money, it cannot replace traditional nursing care because contact with the patient must never be lacking. In my opinion remote nursing support is questionable (…) my illness requires empathy. I wouldn’t say I like the remote nursing role via computer” (P20, M, 46).

#### 3.3.4. Theme 4: The New Relationship Propelled by the Telenursing Care Approach

The new relationship imposed by telenursing was positive for fifteen patients but harmful for others. Three patients were concerned that telenursing could lead to dependency and a lack of privacy in their lives: “I am not sure whether it becomes a psychological addiction… if he’s alone, he hangs up on the phone every 5 min for the loneliness” (P13, M, 77). They perceived the distance relationship with the nurses as ambivalent, oscillating between the need for dependency and the need for distance, closeness, and respect for privacy. Fifteen patients perceived the internet as a chaotic “bubble” in which it was difficult to distinguish correct information from misinformation: “I think it may be useful to have relevant information addressed based on the specific disease, not left to chance, because, often, information is sought indiscriminately via the internet, which obviously cannot replace the doctor nor even the nursing support” (P2, M, 57). It was also used as a tool to compare and be reassured about the expertise of healthcare professionals. The relationships and skills of healthcare professionals emerged as a primary and central tool for the well-being and safety of patients: “The information available online can influence you because it is varied, and it is problematic to find the right one; It depends on where you’re looking. Having direct contact is undoubtedly more attractive” (P19, M, 66).

## 4. Discussion

This phenomenological descriptive qualitative study explored lung cancer and melanoma patients’ perceptions and expectations of telenursing to optimize its implementation and impact on cancer care and clinical practice. Indeed, in-depth knowledge of their perspectives is crucial to implementing personalized, targeted, and patient-accepted care interventions [30]. In particular, the phenomenological approach allowed the authors to explore the sample’s meaning and experience of the phenomenon, in order to grasp its essence [15].

The thematic analysis suggests a higher prevalence of citations from people under 65. In particular, younger people seem to place greater positive expectations on telenursing in terms of its impact on the care relationship (Table 5). At the same time, younger people highlight the possibility that technology can hinder the care relationship, unlike older people (65 years) who do not seem to worry about this aspect, as many studies on the phenomenon suggest [7,31,32]. Despite their greater prevalence in the sample (65% male, 35% female), men highlight the pros and cons of telenursing, revealing greater misunderstandings and concerns about the nature and purpose of telenursing and, at the same time, greater trust in the competence of healthcare professionals, as suggested by a previous study [33]. Indeed, the literature suggests a greater intention to use eHealth tools by men than by women, especially with reference to the perception of their usefulness and the attitude towards their use [30]. With the exception of one patient with melanoma, all patients previously treated with chemotherapy had lung cancer. Undoubtedly, these treatments can cause a significant burden on patients, to which nurses can make an important contribution, both in practical and research fields [34].

Lack of experience leads to misunderstandings, fears, barriers, and preconceptions about the effectiveness of telenursing in cancer care [6,7,35]. Patients describe the internet as a “bubble”. In the size and confusion of the bubble, patients searched for the best facilities to turn to but relied on healthcare professionals for treatment. Similar to previous studies [36], our findings suggest that the nurse–patient relationship is based on critical elements such as mutual trust, effective communication, and the pursuit of common health goals. In line with previous research [3,9,10], respondents considered providing patients with information about the digital approach as a strategic element in promoting its use. Patients involved in this study understand telenursing’s positive and negative aspects, such as reduced waiting times, “always being there”, convenience, the need for training and resources, “cannot replace the human being”, and more: “The home becomes a place where these fragile people can feel better/good. It is possible to work together with the hospital in particularly fragile situations, even from home and remotely, avoiding conducting fragile patients from home to the hospital. Sometimes the nurse’s advice, even remotely, has the same effect as doing it in person, but the fragile patient remains in the safety of his home” (P1, M, 48). The sample involved in this study confirms that information, communication, and empathy were among the best aspects of this approach to care [37]. The fear of losing the relationship with the nurse is a stereotype and a cultural barrier that starkly contrasts the aim of telenursing, which is to enhance care [3]. The interviewees assume that only adults, and not all users, can benefit from this approach, highlighting the complex issue of access to healthcare in line with Levesque’s theory [38] to improve health conditions through effective treatment. The widespread age stereotype does not consider the importance of factors related to health systems, institutions, organizations, and providers in accessing care, as suggested by Levesque [38] and highlighted by seven interviewed patients. In this regard, it is essential to implement multifactorial cross-strategies to improve patients’ use of telenursing, prevent increased health inequalities, and reduce the burden on families and healthcare resources [10].

In line with recent research, interviewees stated that the home is a safe place to care for frail people, including patients with cancer, old patients, and people with chronic conditions [39]: “(Telenursing has the potential) to enable people to learn to use the Internet, including older people, who are always mostly alone when their children grow up and leave home. Maybe some association or someone can teach him to use the Internet to talk to doctors or nurses” (P8, M, 59). In line with recent research [40], the patients interviewed stated that within the home, patients and families build and improve their family and social network according to the cancer experience and the changes it brings. The house becomes a comfortable place of care, but at the same time, patients develop loneliness and experience comfort and distress. Consequently, away from healthcare professionals, the need to use the telenursing approach increases, especially in the advanced stages of cancer, when leaving home for treatment could be more difficult for patients and family members, such as during lockdown [9].

This study highlighted an essential difference in the perception of disease burden between melanoma patients and lung cancer patients. The more significant symptom burden and worsening quality of life may explain this phenomenon in lung cancer patients compared to other cancer patients [41], as shown in Table 4: “That (telenursing) is good because if one has a doubt, is ill and doesn’t physically need nurses, it can undoubtedly be resolved by the phone. If it gets worse, you feel bad, so call. As the disease worsens, you can receive telephone advice: “Do this and then let me know”. So, for the management of side effects, yes (it can be used), because especially during the first chemotherapy (I changed several), I had a lot of side effects. I don’t know about other things, but I don’t think so” (P3, F, 59).

The lack of direct knowledge and experience of the phenomenon has led to an incomplete understanding of the potential and limitations of this approach to managing illness, health, and communication with the healthcare team, as summarized by the emerging themes: “Having a contact number, an email, or an App could be very useful for interfacing with the nursing staff… obviously, it could be other machines, other apps such as video calls, etc. I don’t know exactly how it works, but I think it could be useful” (P11, M, 68).

Regarding the loneliness associated with the digital approach highlighted in the interviews, a recent qualitative study in a non-oncology setting suggests that telenursing interventions improve the nurse–patient relationship [23], allowing nurses to spend more time with patients and fostering a sense of closeness and trust. In this regard, the results of this study could provide interesting information on the implementation of telenursing interventions designed to address patients’ needs, values, and preferences and improve the quality and continuity of care for patients with lung cancer and melanoma.

### 4.1. Limitations

The authors recognize the limitations of the present study. The qualitative approach may reduce the generalizability of the findings. However, Lincoln and Guba’s criteria were used to improve the reliability of the results [18]. The convenience sampling used is certainly not conducive to the generalizability of the results. However, the generalizability of results is not the main purpose of qualitative research, which instead aims to explore the deep experience of the subjects involved and maximize understanding of the phenomenon [19,21]. The lack of patients’ direct experience with telenursing may affect the reliability of their responses but could be helpful in the planning phase of nursing interventions. Exploring the origin of the patients’ urban or rural contexts could have been beneficial for this study’s purpose. The authors are aware that variations in interview length (from 5 to 20 min) may influence the depth of data collected. Shorter interviews may not capture detailed perceptions, leading to underestimations of some themes. Finally, qualitative studies including people with other types of cancer will be crucial to assess the validity of the study results in more heterogeneous and larger populations.

### 4.2. Implications for Practice and Research

The emerging themes suggest a positive attitude of patients towards telenursing, considered a tool to improve communication with the care team and support for patients and families, even in the advanced stages of the disease. In this regard, telenursing can increase the involvement and proactivity of patients in clinical practice, to improve their experience and quality of life, including by providing more technical and practical information useful for managing critical issues related to disease and treatments [42]. According to interviewees, the empathetic relationship with nurses emerges as a pivotal element of their pathways, as opposed to the elusive relationship with physicians, often considered too busy to help patients navigate the chaotic internet “bubble”. Patients’ confidence in telenursing, however, should also be measured through future quantitative studies to detect its impact and cause-and-effect relationship. The interesting sociodemographic differences highlighted by the results of this study, in particular about age and gender, undoubtedly deserve further investigation through heterogeneous research designs and approaches, in order to evaluate their impact on the use of telenursing. The higher prevalence of quotes from surgical patients (Table 5) compared to nonsurgical patients suggests the need for future studies in surgical cancer patients to assess their needs and satisfaction with the use of telenursing. Similarly, the impact of treatments on the use of telenursing certainly represents a further interesting area of development for future nursing research. Providing more information and support to patients and families in accessing and using telecommunications for telenursing could be interesting future research, such as exploring nurses’ opinions and barriers to telenursing to promote a wider dissemination of the model, better use, and stakeholder satisfaction. Finally, the positive expectations patients place on telehealth as end-of-life support offer nurses interesting developments for clinical practice and future research.

## 5. Conclusions

Telenursing is a promising nursing approach for improving patient-centered care, self-care, and patient well-being in cancer care [2,7,43], allowing nurses and patients to interact remotely and improving data collection and connectivity. Although the current literature emphasizes the positive impact of telenursing on continuity of care, the nurse–patient relationship, and the convenience of remote consultations, to the authors’ knowledge, this is the first study to explore the phenomenon in this high-care-load population affected by lung cancer and melanoma. The descriptions of patients with melanoma and lung cancer collected in this study highlighted key factors for improvement, including nurses’ knowledge of the influence of individual, emotional, and relational aspects on patients’ psychophysical and social well-being. Being widely described, concerns about privacy and the importance of the nurse–patient relationship are prevalent in the current literature [36]. However, the results of this study allow researchers to hypothesize that the strength of the nurse–patient relationship allows patients to choose to rely on this innovative approach. In this regard, the nurse–patient relationship is the “fil rouge” of the clinical pathways of the patients interviewed. The themes that emerged from the patient interviews suggest that, despite their inexperience with telenursing, patients with melanoma and lung cancer are largely supportive of its use, recognizing both its potential and its limitations. However, the effective implementation of telenursing interventions depends on many variables, including the available technological infrastructure and the training of nursing staff. As suggested by the emerging themes, reducing the fear of losing the nurse–patient relationship by gaining more information about the care model and its use is a priority for the patients involved [44]. Although this phenomenological research has provided exciting insights into the telenursing approach, further studies are needed to assess its impact on healthcare costs and clinical and nursing outcomes.

## Figures and Tables

**Table 1:** Interview’s guide.

Key Topics	Questions
Health-related internet use	1. Can you tell me about your experience with using the internet to find information about your health?
	2. Did the information you found affect your attitude toward health care personnel?
Previous knowledge and experience with telenursing	3. What does the term “telenursing” make you think about?
	4. Have you ever had experience with remote nursing interventions?
	5. Can you tell me about your experience?
Main expectations, perceptions, and barriers to telenursing	6. Do you feel that telenursing could have an impact on how your disease, health condition, and communication with the care team are managed?
	7. Do you think telenursing could be used for patient education interventions, i.e., for transferring information, knowledge, and skills on therapy, on the most frequent adverse events, on promoting healthy lifestyles, or more?
	8. In your opinion, what is the greatest potential of telenursing?
	9. According to you, what are the main elements that could facilitate or hinder your use of telenursing?

**Table 2:** Sociodemographic and clinical characteristics.

Characteristics	*n*, 20
**Age** (**mean**)	61.7 (42–77)
**Gender**	
Male	13 (65%)
Female	7 (35%)
**Education**	
Primary school	1 (5%)
Middle school	6 (30%)
High school	9 (45%)
Degree	4 (20%)
**Marital status**	
Single	2 (10%)
Married	13 (65%)
Divorced/Separated	2 (10%)
Widow	3 (15%)
**Employment**	
Yes	11 (55%)
No	9 (45%)
**Cohabitation with partner**	
Yes	15 (75%)
No	5 (25%)
**Cohabitation with son/daughter**	
Yes	10 (50%)
No	10 (50%)
**Son/daughter** (**mean**)	1.6 (0–3)
**Cancer**	
Lung cancer	9 (45%)
Melanoma	11 (55%)
**Surgical treatment**	
Yes	11 (55%)
No	9 (45%)
**Chemotherapy**	
Yes	6 (30%)
No	14 (70%)
**Radiotherapy**	
Yes	6 (30%)
No	14 (70%)
**Current treatment**	
Immunotherapy	10 (50%)
Chemotherapy	4 (20%)
Radiotherapy	1 (5%)
Staging	3 (15%)
None	2 (10%)

**Table 3:** Patient and interview information.

Patient	Age	Sex	Type of Cancer	Prevalence in the Themes	Length of the Interview
P1	48	Male	Melanoma	5	20 min
P2	57	Male	Melanoma	4	11 min
P9	60	Female	Melanoma	6	5 min
P10	61	Male	Melanoma	5	5 min
P11	68	Male	Melanoma	6	5 min
P12	61	Female	Melanoma	3	5 min
P13	77	Male	Melanoma	6	8 min
P14	44	Female	Melanoma	3	13 min
P15	42	Female	Melanoma	4	6 min
P16	48	Female	Melanoma	4	8 min
P17	57	Male	Melanoma	3	5 min
P3	59	Female	Lung	3	5 min
P4	77	Male	Lung	3	6 min
P5	66	Male	Lung	3	7 min
P6	73	Male	Lung	5	6 min
P7	76	Male	Lung	7	8 min
P8	59	Male	Lung	8	6 min
P18	75	Female	Lung	4	5 min
P19	66	Male	Lung	3	6 min
P20	46	Male	Lung	7	11 min

**Table 4:** Prevalence of themes and sub-themes by cancer.

Theme	Sub-Theme	Melanoma	Lung Cancer
Lack of experience can lead to mistakes	Preconception and misunderstanding	P2, P9, P10, P11, P13, P15, P17	P4, P6, P7, P8, P18, P19, P20
	Someone for you beyond the phone	P9, P11, P13	P3, P6, P7, P8, P18, P19
	Strengths and weaknesses of the connection	P2, P10, P11, P15, P16, P17	P3, P4, P5, P6, P7, P8
Home is the place where frail people are cared for	A bridge between home and care	P1, P13, P16	P4, P7
	Frailties within fragility: age-related barriers	P10, P12, P13, P15	P7, P8, P20
	The family grows in illness	1, 14	P5, P6, P18, P20
	Conditions of psychophysical fragility require support	P1, P9, P11, P12, P14	P5, P7, P20
Telenursing is the link between opportunity and inaccessibility	Telenursing innovates the care approach	P1, P20	P3, P8, P9, P10, P14, P15, P16
	Technology hinders the therapeutic alliance	P2, P9	P8, P20
The new relationship propelled by the telenursing care approach	The caring relationship with the nurse: a choice between dependence and detachment	P11, P13	P8
	From the chaotic bubble of the internet to having trust in healthcare professionals	P1, P2, P9, P10, P11, P12, P13, P16, P17	P6, P7, P8, P18, P19, P20

**Table 5:** Prevalence of themes and sub-themes by cancer, Age, Gender, Surgical Treatment, and Current Treatment.

	Theme	Sub-Theme	Prevalence by Cancer	Prevalence by Age	Prevalence by Gender	Prevalence by Surgical Treatment	Prevalence by Current Treatment
The nurse–patient relationship is the cornerstone of the telenursing care approach	Lack of experience can lead to mistakes	Preconception and misunderstanding	7 Melanoma	7 < 65 y.o.	3 F	7 Yes	7 immunotherapy
							2 chemotherapy
			7 Lung Cancer	7 ≥ 65 y.o.	11 M	7 Not	1 radiotherapy
							4 staging/F.U.
		Someone for you beyond the phone	3 Melanoma	3 < 65 y.o.	3 F	3 Yes	3 immunotherapy
							2 chemotherapy
			6 Lung Cancer	6 ≥ 65 y.o.	6 M	6 Not	1 radiotherapy
							3 staging/F.U.
		Strengths and weaknesses of the connection	6 Melanoma	7 < 65 y.o.	3 F	7 Yes	5 immunotherapy
							4 chemotherapy
			6 Lung Cancer	5 ≥ 65 y.o.	9 M	5 Not	1 radiotherapy
							2 staging/F.U.
	Home is the place where frail people are cared for	A bridge between home and care	3 Melanoma	2 < 65 y.o.	1 F	3 Yes	3 immunotherapy
							1 chemotherapy
			2 Lung Cancer	3 ≥ 65 y.o.	4 M	2 Not	1 radiotherapy
							0 staging/F.U.
		Frailties within fragility: age-related barriers	4 Melanoma	5 < 65 y.o.	2 F	4 Yes	4 immunotherapy
							1 chemotherapy
			3 Lung Cancer	2 ≥ 65 y.o.	5 M	3 Not	1 radiotherapy
							1 staging/F.U.
		The family grows in illness	2 Melanoma	3 < 65 y.o.	2 F	3 Yes	2 immunotherapy
							1 chemotherapy
			4 Lung Cancer	3 ≥ 65 y.o.	4 M	3 Not	0 radiotherapy
							3 staging/F.U.
		Conditions of psychophysical fragility require support	5 Melanoma	5 < 65 y.o.	3 F	5 Yes	5 immunotherapy
							1 chemotherapy
			3 Lung Cancer	3 ≥ 65 y.o.	5 M	3 Not	1 radiotherapy
							1 staging/F.U.
	Telenursing is the link between opportunity and inaccessibility	Telenursing innovates the care approach	2 Melanoma	9 < 65 y.o.	5 F	6 Yes	6 immunotherapy
							2 chemotherapy
			7 Lung Cancer	0 ≥ 65 y.o.	4 M	3 Not	0 radiotherapy
							1 staging/F.U.
		Technology hinders the therapeutic alliance	2 Melanoma	4 < 65 y.o.	1 F	2 Yes	1 immunotherapy
							1 chemotherapy
			2 Lung Cancer	0 ≥ 65 y.o.	3 M	2 Not	0 radiotherapy
							2 staging/F.U.
	The new relationship propelled by the telenursing care approach	The caring relationship with the nurse: a choice between dependence and detachment	2 Melanoma	1 < 65 y.o.	0 F	2 Yes	2 immunotherapy
							1 chemotherapy
			1 Lung Cancer	2 ≥ 65 y.o.	3 M	1 Not	0 radiotherapy
							0 staging/F.U.
		From the chaotic bubble of the internet to having trust in healthcare professionals	9 Melanoma	9 < 65 y.o.	4 F	8 Yes	8 immunotherapy
							1 chemotherapy
			6 Lung Cancer	6 ≥ 65 y.o.	11 M	7 Not	1 radiotherapy
							5 staging/F.U.

Note: y.o. = Years Old; F = Female; M = Male; F.U. = Follow-up.

## Data Availability

The data supporting this study’s findings are available from the corresponding author upon reasonable request.

## Supplementary Materials

The following supporting information can be downloaded at: https://www.mdpi.com/article/10.3390/nursrep14040198/s1, Table S1: COREQ (Consolidated Criteria for Reporting Qualitative Research) Checklist; Table S2: Quotes from themes and sub-themes.

## Public Involvement Statement

Patients were involved in the data collection phase of the study.

## Guidelines and Standards Statement

Consolidated Criteria for Reporting Qualitative Research (COREQ) guidelines were used for the reporting [17].
