# Supplementary material for: Perceptions and Expectations of Patients with Lung Cancer and Melanoma about the Telenursing Approach: A Phenomenological Study
Source: Nurs Rep. 2024 Sep 27;14(4):2680–94. doi: 10.3390/nursrep14040198 (PMC11503421; doi:10.3390/nursrep14040198)
Supplement: Supplementary file 1 [file nursrep-14-00198-s001.zip › nursrep-3110532-supplementary.pdf]

Table S1. COREQ (COnsolidated criteria for REporting Qualitative research) Checklist

Topic	Item No.	Guide Questions/Description	Reported on Page No.
**Domain 1: Research team and reflexivity**			
Personal characteristics			
Interviewer/facilitator	1	Which author/s conducted the interview or focus group?	6-7
Credentials	2	What were the researcher's credentials? E.g. PhD, MD	6-7
Occupation	3	What was their occupation at the time of the study?	title page
Gender	4	Was the researcher male or female?	title page
Experience and training	5	What experience or training did the researcher have?	6-7
Relationship with participants			
Relationship established	6	Was a relationship established prior to study commencement?	8
Participant knowledge of the interviewer	7	What did the participants know about the researcher? e.g. personal goals, reasons for doing the research	6-8
Interviewer characteristics	8	What characteristics were reported about the inter viewer/facilitator? e.g. Bias, assumptions, reasons and interests in the research topic	7-8
**Domain 2: Study design**			
Theoretical framework			
Methodological orientation and Theory	9	What methodological orientation was stated to underpin the study? e.g. grounded theory, discourse analysis, ethnography, phenomenology, content analysis	6-8
Participant selection			
Sampling	10	How were participants selected? e.g. purposive, convenience, consecutive, snowball	6-8
Method of approach	11	How were participants approached? e.g. face-to-face, telephone, mail, email	6-8
Sample size	12	How many participants were in the study?	9
Non-participation	13	How many people refused to participate or dropped out? Reasons?	9
Setting			
Setting of data collection	14	Where was the data collected? e.g. home, clinic, workplace	6-7
Presence of non-participants	15	Was anyone else present besides the participants and researchers?	7
Description of sample	16	What are the important characteristics of the sample? e.g. demographic data, date	Tables 2
Data collection			
Interview guide	17	Were questions, prompts, guides provided by the authors? Was it pilot tested?	7
Repeat interviews	18	Were repeat interviews carried out? If yes, how many?	9
Audio/visual recording	19	Did the research use audio or visual recording to collect the data?	6-7
Field notes	20	Were field notes made during and/or after the interview or focus group?	6-8
Duration	21	What was the duration of the interviews or focus group?	7; Table 3
Data saturation	22	Was data saturation discussed?	7-9
Transcripts returned	23	Were transcripts returned to participants for comment and/or	N/A

Topic	Item No.	Guide Questions/Description	Reported on Page No.
		correction?	
**Domain 3: analysis and findings**			
Data analysis			
Number of data coders	24	How many data coders coded the data?	7-8
Description of the coding tree	25	Did authors provide a description of the coding tree?	9-11
Derivation of themes	26	Were themes identified in advance or derived from the data?	7-9
Software	27	What software, if applicable, was used to manage the data?	8
Participant checking	28	Did participants provide feedback on the findings?	N/A
Reporting			
Quotations presented	29	Were participant quotations presented to illustrate the themes/findings? Was each quotation identified? e.g. participant number	9-11; Supplementary eTables
Data and findings consistent	30	Was there consistency between the data presented and the findings?	9-13
Clarity of major themes	31	Were major themes clearly presented in the findings?	9-13
Clarity of minor themes	32	Is there a description of diverse cases or discussion of minor themes?	9-13

Developed from: Tong A, Sainsbury P, Craig J. Consolidated criteria for reporting qualitative research (COREQ): a 32-item checklist for interviews and focus groups. *International Journal for Quality in Health Care*. 2007. Volume 19, Number 6: pp. 349 – 357

Table S2: Quotes from Themes and Sub-themes

General Theme	Sub-themes		Quotes
General Theme: The nurse-patient relationship is the cornerstone of the telenursing care approach	**Lack of experience can lead to mistakes**	*Preconception and misunderstanding (P2; P4; P6; P7; P8; P9, P10; P11; P13; P15; P17; P18; P19; P20.)*	"I first heard this term when I was enrolled in this study. I have heard of telemedicine from some television programs talk about it. However, I heard about telenursing for the first time when I got involved in this study" **(P2, M, 57)**. "This is the first time I've heard this! It seems like a new TV show to me (laughs). I think it's help from the nurses from home. I have some doubts, and I need to understand precisely what telenursing could be. Of course, I hope it is for the patient's benefit and can benefit him, but I need to know the precise answer" **(P4, M, 77)**. I once called a nurse who suggested a pharmacy. He almost killed me by putting the wrong bladder catheter in me. She came to my house and changed it very severely" **(P6, M, 73)**. I used telehealth when I once called a nurse to insert a bladder catheter. He made a mistake by changing it badly, and it almost killed me. 100% negative" **(P7, M, 76)**. "During the COVID pandemic, I have had video calls with doctors. Who knows if it's the same for telenursing, but this helped reassure me. If I understand correctly, what telenursing could do is an interface between the nurse and a different hospital to which I may have turned for an illness. (Telenursing has the potential) to enable people to learn to use the Internet, including older people, who are always mostly alone when their children grow up and leave home. Maybe some association or someone can teach him to use the Internet to talk to doctors or nurses" **(P8, M, 59)**. "Yes, for example, about eating and nutrition, did this thing hurt me? Yes, no, it has nothing to do with nutrition, or it has something to do with it, so it's better to avoid it. It could be helpful for the most trivial of daily problems" **(P9, F, 60)**. "This is the first time I've heard this word. I have no idea about it" **(P10, M, 61)**. "Having a contact number, an email, or an App could be very useful for interfacing with the nursing staff... obviously, it could be other machines, other apps such as video calls, etc. I don't know exactly how it works, but I think it could be useful" **(P11, M, 68)**. "I don't know what aspects or topics telenursing can be used for, or at least I don't imagine it now. Maybe some advice for people who can't leave home" **(P13, M, 77)**. "It could be beneficial, especially in case of need. As long as things are going well, it wouldn't be helpful, but when there was a problem, a doubt, anything... it would help. Even just direct communication with the department to inform them that a patient cannot come for therapy is ill, has a doubt about a symptom, or other" **(P15, F, 42)**. "Something Information Technology that can somehow put a patient in contact through the computer, through modern systems, with people specialised in the nursing field. I haven't had any significant side effects from the actual treatment, but if I had had any, a service of this kind could be beneficial...Help people to act correctly to avoid, for example, making dangerous mixtures of drugs" **(P17, M, 57)**. "Telenursing makes me think of American nurses. I think it could be a very beneficial service but I'm not sure what it is. I don't know, but I think it's suitable for patients." **(P18, D, 75)**. "It is certainly something more to help patients interface with unreachable doctors or facilities. It can offer a quick answer, but at the moment, I can't say what it could be used for. Maybe it can avoid clogging up the emergency room" **(P19, M, 66)**. "But I just ask one question: Why contact a nurse, not a doctor, if you have a medical doubt? I would ask a nurse for medication, injections, and something else that I wouldn't ask a doctor. To find out if it is better to take one drug or another or to say what happened to me, culturally, we are used to asking the doctor and not the nurse" **(P20, M, 46)**.

		Someone for you beyond the phone (P3; P6; P7; P8; P9; P11, P13; P18; P19.)	"That (telenursing) is good because if one has a doubt, is ill and doesn't physically need nurses, it can undoubtedly be resolved by the phone If it gets worse, you feel bad, so call. As the disease worsens, you can receive telephone advice: "Do this and then let me know". So, for the management of side effects, yes (it can be used), because especially during the first chemotherapy (I changed several), I had a lot of side effects. I don't know about other things, but I don't think so" **(P3, F, 59)**. "You know who to call in times of need. Today, if you don't have acquaintances when you're sick, you call a friend. Instead, you immediately contact the people who can indicate or direct you. Maybe you have a problem, and he will solve it by phoning" **(P6, M, 73)**. "You can call if you have something or feel unwell; it's resolved. Timeliness. However, let's say it's a useful way not always applicable. Dealing with someone with an expert on the other side of the computer" **(P7, M, 76)**. "The doctor is only sometimes available for a problem, so you may always have someone to call. In short, the nurse can help you explain more things than the doctor. The doctor is always a little busier, right? No one discriminates against the nurse, but the nurse lives in the hospital daily, while the doctor is there for 5/6 hours and then goes home." **(P8, M, 59)**. "It reassures me. If I have any doubts about the disease, I can call, and I will remove the doubt, anxiety and fear. Promptness means picking up the phone, calling, telling you what to do, and they'll tell you straight away; I hope so" **(P9, F, 60)**. "I think it can help because very often it happens that you have doubts, uncertainties, pain, or something, and you would like to ask for information, but obviously, you can't talk to the doctor all the time. Often, you don't even have the doctor's number, and so if you send the email, you have to wait for him to reply because they rightly have things to do with all these patients. It's as if it were an always active number to call **(P11, M, 68).** "An older person may have some problems with technology, so I was referring more to the use of the telephone because we do not have the ease and immediacy that young people have in using the computer" **(P13, M, 77)**. I think it's good to call a nurse for a good word when I'm sick. I think it could be a very beneficial service, but I'm not sure what it is. I don't know, but I think it's suitable for patients **(P18, D, 75)**. Surely they could help me when it is not possible to contact a doctor or the facility. Having someone who can give you a quick answer is definitely great. But there should be someone who can answer the phone **(P19, M, 66)**.

		Strengths and weaknesses of the connection (P2; P3; P4; P5; P6; P7; P8; P10; P11; P15; P16; P17.)	"What can hinder is misinformation, whereby the patient is not informed about the existence and methods of access...then it depends on the tools available" **(P2, M, 57)**. "I don't see any obstacle to using telenursing; I'm in favour of it. Indeed, the patient must not have significant cognitive impairments" **(P3, F, 59)**. "Patients often ask themselves: "But will this do us any good?". I don't know how this can improve patients' health or be useful to them. But I don't think it can make your health worse. I think it could be a futuristic approach, offering future health benefits in short" **(P4, M, 77)**. "I don't care about my illness, I don't go on the internet, I don't care. I am 66 years old, and this progress is unthinkable for me. Today, it could be helpful, and whether we like it or not, technology is everywhere. However, we will see how telenursing will be used because everything can get dirty" **(P5, M, 66)**. "That's always a good thing, but I would never use it by nature. It already gives me trouble talking to a doctor. I think there can be no obstacles, except if you can't connect. Everything is favourable for patients because they can always talk to competent people" **(P6, M, 73)**. (In my opinion, it could not be helpful for patient education) because everyone must have their skills. I have worked for fifty years and know that competence is the first thing: if there is no competence, there are no results. No obstacles. It can be used as needed" **(P7, M, 76)**. The only obstacle could be the lack of Internet **(P8, M, 59)**. "In my opinion, having an immediate discussion with people who can give answers, is undoubtedly a positive thing. I honestly think it could be a positive tool for patients, and I don't give up any obstacles" **(P10, M, 61)**. "It's a great way to connect (…) having a contact number or an email or an app where you can communicate with the nursing staff" **(P11, M, 68)**. "Side effects to discuss with the nursing staff and be reassured about its severity" **(P15, F, 42)**. "I believe everything that is informative can easily be replaced via the internet. I am very much in favour of such an attitude. I don't see any obstacle other than the availability of an Internet connection" **(P16, F, 48)**. "Sometimes, I need a better connection. Very often, the line, despite having been improved, is weak; sometimes, the connection might be more optimal. Having an optimal connection to the Internet, that's all" **(P17, M, 57)**.
	**Home is the place where frail people are cared for**	*A bridge between home and care (P1; P4; P7; P13; P16.)*	"The home becomes a place where these fragile people can feel better/good. It is possible to work together with the hospital in particularly fragile situations, even from home and remotely, avoiding conducting fragile patients from home to the hospital. Sometimes the nurse's advice, even remotely, has the same effect as doing it in person, but the fragile patient remains in the safety of his home **(P1, M, 48)**". "I believe that (telenursing) can benefit the patient's health. People have been cured in hospitals, but it is also true that telenursing can be helpful from home. Of course, patients often sit down at the table and think: but will this do us any good? Do you think this will be useful?" **(P4, M, 77)**. "If it is not a serious health problem, it could also be solved this way at home. Surely, if I need to have some tests, I need to leave my home and come to the hospital" **(P7, M, 76)**. "Unless the therapy can also be administered at home, the nurse comes, and carries out the therapy, it would be theoretically possible. However, contact with the doctor would still be necessary, if you don't mind. Indeed, given how the visits are carried out, everything would stay the same if they were done remotely" **(P13, M, 77)**. "Taking care of me is better at home...Telenursing could offer more significant information without increasing the workload and the burden of nurses who could carry out more practical activities in person, such as medication, which is impossible remotely. I can move and go out, and I'm not stuck at home, so I don't need telenursing now. However, it could be helpful if I find myself on an island or in an area that is difficult to reach" **(P16, F, 48)**.

		Frailties within fragility: age-related barriers (P7; P8; P10; P12; P13; P15; P20.)	"I am 77 years old and old-fashioned. I don't look for information on the Internet" **(P7, M, 76)**. "(Telenursing has the potential) to enable people to learn to use the Internet, including older people, who are always mostly alone when their children grow up and leave home. Maybe some association or someone can teach him to use the Internet to talk to doctors or nurses" **(P8, M, 59). "**It will undoubtedly be a little more problematic for older people, but they have to get used to everything a little, perhaps with the help of a relative or someone who assists them" **(P10, M, 61).** "I've never looked because there's always a bit of fear with the internet, and I'm old-fashioned" **(P12, F, 61).** "My daughter, who is young, as soon as she found out about the melanoma, started looking for everything that corresponded to it, then she told me. An older person may have some problems with technology, so I was referring more to the use of the telephone because we do not have the ease and immediacy that young people have in using the computer" **(P13, M, 77)**. "I am pretty young, and I can establish a connection with healthcare professionals as I want. An older person without an internet connection 24 hours a day could have difficulty using telenursing, but it could be a helpful resource" **(P15, F, 42).** Unfortunately, the topic of computerisation clashes a bit with a generational problem. I think about how I could use such a service and how my mother could use it" **(P20, M, 46)**.
		The family grows in illness (P1; P5; P6; P14; P18; P20)	"(The illness) upsets the whole family. The family is attentive to the patient's needs. The patient must feel this silent work around him....The daughters went into crisis because they did not grow from that point of view, delegating the care and management of their mother to a third person. However, unlike the girl, they did not grow up with the disease and did not acquire basic professional skills that could have been useful during the disease" **(P1, M, 48)**."More members of my family suffer from it than I do" **(P5, M, 66)**. "I only talk to my son or my wife, and they are the ones who get the information. It already gives me trouble talking to a doctor **(P6, M, 73)**. "The disease makes you alone, even within your family. Sometimes, many people are close to you, but they don't know how to help you. An external person could also help family members who don't know what or how to say or how to behave **(P14, F, 44)**. If something happens to me, I ask my grandchildren, my daughter, my family **(P18, D, 75)**. If you have a friend who is a nurse, if you have a doubt you always ask him, and it can help the whole family. It happened to my family before this illness with the wife of a friend of mine who is a nurse in obstetrics. "Silvia, I need an injection. Can you help me?". But there has already been a step further: a personal relationship". **(P20, M, 46)**

		Conditions of psychophysical fragility require support (P1; P5; P7; P9; P11; P12; P14; P20)	"It is possible to work together with the hospital in particularly fragile situations, even from home and remotely, avoiding conducting fragile patients from home to the hospital. You can also grow by acquiring some little skills, compared to a nurse... so that, when the nurse tells you what to do, on the other side there is an active person ready to understand what the nurse tells him" **(P1, M, 48)**. "People want to talk about their illness, so I believe that (telenursing) can be a suitable response to this need" **(P5, M, 66)**. "It could also be resolved (with telenursing) if it is not a severe event. If I need to have some tests, I need to leave my home and come to the hospital" **(P7, M, 76)**. "This could be a relief for ill people who find that they have something terrible, don't know what's behind it, and don't have other people to talk to. It could also provide moral support to people because, in the illness, you feel fragile and afraid, but in that moment, an answer comes immediately and takes you away from fear and anxiety. They would be our angels watching over us" **(P9, F, 60)**. "Caring may seem silly, but it is the main thing for patients. This is different from my case, but sometimes I think that you can have problems just finding some drugs. Sometimes, you don't know who to ask or turn to for pain during the night, and you can't go to the doctor's office, and you can have 24-hour support. If you can have 24/7 support, even if I don't know how it works, it's suitable for patients **(P11, M, 68)**. "I didn't look for information because my husband did everything. I couldn't do anything; the whole part was blocked, so my husband cared for everything. But so far, I've been fine...Help people when they feel a little alone; maybe a word from an expert could help them overcome the moment" **(P12, F, 61)**. "The illness makes you feel very lonely even within your own family...If the patient has closed in on himself, you cannot do anything; you will never be able to knock down that wall, but I hold my hand to you, even if you are not ready to welcome it. Knowing that someone can listen to you alone makes you feel better, regardless of whether they call you or make a video call" **(P14, F, 44)**. "Not all people are ready to learn via video, so if there is a poor service performance, can we say it didn't work because telenursing doesn't work? Or because the nurse wasn't good? Or because the patient was not receptive? Who decides? What are the evaluation criteria?"**(P20, M, 46)**.
	**Telenursing is the link between opportunity and inaccessibility**	*Telenursing innovates the care approach (P1; P3; P8; P9; P10; P14; P15; P16; P20.)*	"It's an extra weapon…. That bow is really heavy, instead we need to lighten it...(With telenursing) we all take responsibility for the care, and in any case, we end up having much less impact on the hospital, transportation, parking and everything, because the care moves to the person, and the nurse arrives in people's homes. " **(P1, M, 48)**. "Even from a human point of view, perhaps the patient says: "I feel like this; it has never happened to me; what can I do?" Then, the nurse interacts with the patient by directing the best path to take" **(P3, F, 59)**. "I think that (even remotely) the nurse can help you explain more things than the doctor says. The doctor is always a little busier" **(P8, M, 59)**. "I don't have any more doubt, nor fear or anxiety" **(P9, F, 60)**. "Constantly interacting immediately with people who can give answers is undoubtedly a positive thing. Nowadays, interactive meetings remotely are almost the same as meeting physically, so I find this to be a positive thing" **(P10, M, 61)**. "I have always hated telemedicine, but in this case, perhaps it would be the true meaning of telemedicine. A project like this doesn't exist anywhere (...) it is a distant reality" **(P14, F, 44). "**Mainly moral support, but it could also be the security of knowing someone is there for you" **(P15, F, 42)**. "A person dedicated to you at that moment has a calming effect, and you do not get stressed by the long wait. We could be dealing with less dissatisfaction among healthcare personnel, overwhelmed by too many face-to-face activities to which consultancy must also be added... today the patient often receives a correct but hasty message, delivered by nurses out of a sense of duty but tiring. Instead of figures dedicated only to this, in my opinion, they would allow for more outstanding patient care" **(P16, F, 48)**. "One of the biggest benefits this service provides is its speed" **(P20, M, 46)**.

		Technology hinders the therapeutic alliance (P2; P8; P9; P20.)	“It cannot replace either doctors or the nurses” **(P2, M, 57)**. “I always prefer to talk to a human being rather than to a machine” **(P8, M, 59).** “Maybe the phone rings and no one ever answers, and then I become more anxious, and my anxiety level increases even more. I hope it is a good service that everyone can use, and not just some people” **(P9, F, 60).** “I have some doubts caused by my perception of the nature of the nurse's work compared to that of the doctor. I never expect great empathy from the doctor, I hope it from a nurse, precisely on a human level and the telematic relationship is cold, by nature. It must not become a replacement. Direct contact with patients must never be lacking in patient care...Even being Italian could be a cultural problem; that is, if this service allows you to save money, it cannot replace traditional nursing care because contact with the patient must never be lacking. In my opinion remote nursing support is questionable (...) my illness requires empathy. I wouldn't say I like the remote nursing role via computer” **(P20, M, 46)**.
	**The new relationship propelled by the telenursing care approach**	*The caring relationship with the nurse: a choice between dependence and detachment (P8; P11; P13.)*	"I don't think anyone could force me to use it or not if I don't want to" **(P8, M, 59)**. “Respect for the patient's privacy is important because it can happen that someone doesn't know, and avoiding it is better” **(P11, M, 68)**. “I am not sure whether it becomes a psychological addiction... if he's alone, he hangs up on the phone every 5 minutes for the loneliness” **(P13, M, 77)**.
		From the chaotic bubble of the internet to having trust in healthcare professionals (P1; P2; P6; P7; P8; P9; P10; P11; P12; 13; P16; P17; P18; P19; P20.)	"I have always sought a direct relationship with healthcare personnel, not letting myself be distracted by the internet. Yes, I looked for it (the information) out of pure curiosity, but then, I have always relied on healthcare professionals" **(P1, M, 48)**."I think it may be useful to have relevant information addressed based on the specific disease, not left to chance, because, often, information is sought indiscriminately via the internet, which obviously cannot replace the doctor nor even the nursing support" **(P2, M, 57)**. "I haven't done any research because I'm scared. The Internet generalises information while the nurse gives you a competent answer. (Telenursing) always allows you to talk to people and not just go there and read" **(P6, M, 73)**. "Since I am in an excellent cancer center, it is useless to search on the Internet. I rely on them" **(P7, M, 73)**. "When I heard about the disease, I looked for information. But I immediately deleted from my memory the information from the Internet, which I believe is minor. I always prefer talking to a human being rather than a machine" **(P8, M, 59)**. "As soon as they told me about my illness, I immediately asked the Internet because it is the only way to get information, and there I found everything and more. It was a considerable shock because it was agitating, not knowing exactly what was happening to me, what I would face, and what would become of my life. Therefore, I was terrified" **(P9, F, 60)**. “I asked the internet but not proactively; it was just to look for some information. I trust doctors much more than I do the internet. I read something, but then I rely above all on the doctor” **(P10, M, 61)**. "I have never looked online about my illness because I know that information can be different. To avoid worrying unnecessarily, I prefer to speak directly to my doctor" **(P11, M, 68)**. "I've never looked because there's always a bit of fear with the internet, and I'm old-fashioned" **(P12, F, 61)**. "My daughter, who is young, as soon as she found out about the melanoma, started looking for everything that corresponded to it, then she told me. I received information indirectly, but I never asked to the internet" **(P13, M, 77)**. "As the daughter of a doctor, I usually avoid looking for health information on the Internet because it often offers unreliable information...they always explained everything to me in the hospital" **(P16, F, 48)**. “I have never researched my illness on the Internet because all the sites talk about recovered people or living corpses. The information on the Internet was totally contradictory” **(P17, M, 57)**. "I have never looked for information on the Internet about my illness because I don't want to waste time" **(P18, D, 75)**. "The information available online can influence you because it is varied, and it is problematic to find the right one; It depends on where you're looking. Having direct contact is undoubtedly more attractive" **(P19, M, 66)**. "I'm a person who believes that everyone should do their job. So spending two hours on the internet doesn't make me a doctor...The problem of the reliability of sources on the internet is probably the plague of the third millennium" **(P20, M,46)**.
